# Evaluation of the accuracy of cone-beam computed tomography image segmentation of isolated tooth roots based on the dynamic threshold method

**DOI:** 10.1186/s12903-023-03423-y

**Published:** 2023-10-13

**Authors:** Sha Su, Yu-meng Liu, Li-ping Zhan, Si-yuan Gao, Cai He, Qing Zhang, Xiao-feng Huang

**Affiliations:** 1 Department of Stomatology, Beijing Friendship Hospital, Capital Medical University, 95 Yong ’an Road, Xicheng District, Beijing, 100050 China; 2 Department of General surgery, Beijing Huaxin Hospital, the First Affiliated Hospital of Tsinghua University, Beijing, 100016 China

**Keywords:** Cone-beam computed tomography, Root surface area, Threshold-based method, Tooth root segmentation

## Abstract

**Objective:**

Accurate quantification of the root surface area (RSA) plays a decisive role in the advancement of periodontal, orthodontic, and restorative treatment modalities. In this study, we aimed to develop a dynamic threshold-based computer-aided system for segmentation and calculation of the RSA of isolated teeth on cone-beam computed tomography (CBCT) and to assess the accuracy of the measured data.

**Method:**

We selected 24 teeth to be extracted, including single-rooted and multi-rooted teeth, from 22 patients who required tooth extraction. In the experimental group, we scanned 24 isolated teeth using CBCT with a voxel size of 0.3 mm. We designed a computer-aided system based on a personalized dynamic threshold algorithm to automatically segment the roots of 24 isolated teeth in CBCT images and calculate the RSA. In the control group, we employed digital intraoral scanner devices to perform optical scanning on 24 isolated teeth and subsequently manually segmented the roots using 3-matic software to calculate the RSA. We used the paired t-test (*P* < 0.05) and Bland-Altman plots to analyze the consistency of the two measurement methods.

**Results:**

The results of the paired t-test showed that there was no significant difference in the RSAs obtained using the dynamic threshold method and the optical scanning image reconstruction (t = 1.005, *P* = 0.325 > 0.05). As per the Bland-Altman plot, the results were evenly distributed within the region of ± 1.96 standard deviations of the mean, with no increasing or decreasing trends and good consistency.

**Conclusion:**

In this study, we designed a computer-aided root segmentation system based on a personalized dynamic threshold algorithm to automatically segment the roots of isolated teeth in CBCT images with a voxel size of 0.3 mm. We found that the RSA calculated using this approach was highly accurate, and a voxel of 0.3 mm in size could accurately display the surface area data in CBCT images. Overall, our findings in this study provide a foundation for future work on accurate automatic segmentation of tooth roots in full-mouth CBCT images and the computation of RSA.

## Introduction

The accurate measurement of root geometry facilitates the graded diagnosis of periodontal disease, the assessment of root resorption during orthodontic treatment, and the development of restorative plans [1, 2]. In the past 20 years, devices such as the optical scanner, [3] and techniques such as micro-computed tomography (micro-CT) [4] and cone-beam computed tomography (CBCT) [5] in conjunction with 3D reconstruction software such as 3-matic and Mimics have replaced traditional root geometric measurement methods [6, 7]. Intraoral scanners (IOS) are considered highly accurate in comparison to oral plaster models when scanning short-span areas such as a tooth or a bridge up to half an arch [8–12]. The accuracy of area measurement using optical scanners has also been verified in skin measurement tests [13].

CBCT compensates for the disadvantage of the scanning method, which is that only the crown can be scanned. It also provides significant dose reductions of between 98.5% and 76.2% when compared to the patient dose reported for maxillofacial imaging by conventional CT (approximately 2000 mSv) [14, 15]. Initial research on the accuracy of CBCT focused on the evaluation of linear [16, 17] or volumetric data, [18, 19] and was shown to be highly accurate with regard to alveolar bone defects and root length of varying voxel sizes (0.125–0.40 mm) [20, 21]. However, there are only a few studies that compare the accuracy of RSA data in CBCT images of human teeth [5, 22].

In addition, segmenting the root from the crown of the tooth is a challenge in CBCT image segmentation. Currently, irrespective of purely manual or semi-automatic segmentation procedures, the structure of interest needs to be depicted based on adjacent regions by the human eye, and this causes subjectivity in segmentation [23] and leads to observer fatigue, which affects the reliability of the technique [24, 25].

Li et al [26] proposed an automatic root segmentation method using the U-Net neural network. However, they do not mention how to identify the cementoenamel junction (CEJ) of the tooth. The CEJ is the anatomical boundary between the enamel-covered crown and the cementum-covered root [27]. However, this structure on the surface of the tooth appears smoothly curved, without any anatomical markings of protrusions or depressions; there is only a difference in threshold values in the images at this boundary. Many previous studies have reported the disadvantages of threshold segmentation [28, 29]. Grayscale values in CBCT devices are not standardized because of variations in imaging protocols and specified automated exposure settings [30]. Moreover, the grayscale value may change for objects with the same radiation density, depending on their relative positions [31].

Therefore, in this study, we sought to design a new automatic root segmentation method based on personalized dynamic thresholds to segment isolated tooth roots in CBCT images and calculate RSA. At the same time, we compared the accuracy of CBCT area data of 0.3 mm voxel size with the measurement results of RSA obtained using an intraoral optical scanner.

## Materials and methods

This study was approved by the Research Ethics Committee of the Beijing Friendship Hospital, Capital Medical University (No. 2021-P-130-01) and was conducted in accordance with the principles of the Declaration of Helsinki.

### Data acquisition for the tooth segmentation model

We estimated the sample size using prior studies and power analysis, with a calculation method of α = 0.05, a power of 0.90, and a mean pairing difference value of 10 mm. It was determined that 22 samples were required with 20% changes in the effect size to represent a significant difference in RSA values.

For the experimental group, we selected 22 patients who underwent extraction for orthodontics, chronic periodontitis, and impacted third molars in the outpatient clinic of the Beijing Friendship Hospital, Capital Medical University, between July 2022 and September 2022. A total of 24 affected teeth (19 third molars, 4 first premolars, and 1 incisor tooth) were extracted. The extracted teeth were immediately immersed in a 5.25% sodium hypochlorite solution for 30 min. The residual soft tissues attached to the root surface were then gently removed without damaging the tooth tissues. All teeth were intact and free of caries, defects, or root fractures and included ten single-rooted, nine twin-rooted, and five multi-rooted teeth.

We placed each isolated tooth in a transparent, square plastic box. We placed the tooth and the box in the CBCT scan area. We adjusted the horizontal and vertical positions of the scan area such that the tooth was centered in the CBCT scan area and then performed the scan.

CBCT images were acquired from a CBCT scanner (NewTom CT, Cefla QR Verona, Verona, Italy) with a field of view of 12 × 8 cm^2^, voxel spacing of 0.3 mm (an isotropic voxel size of 0.3 mm), exposure parameters of 110 kV, 2.1–4.4 mA (depending on subject size), and 3.6 s. CBCT scanning was rotated 360°. The 2D images were obtained by reformatting along the plane perpendicular to those of the axial (or tilted) slice. The image grayscale depth was 8 bits. The output CBCT images and corresponding parameters were stored in the Digital Imaging and Communications in Medicine (DICOM) file format for further analysis.

**Fig. 1:**
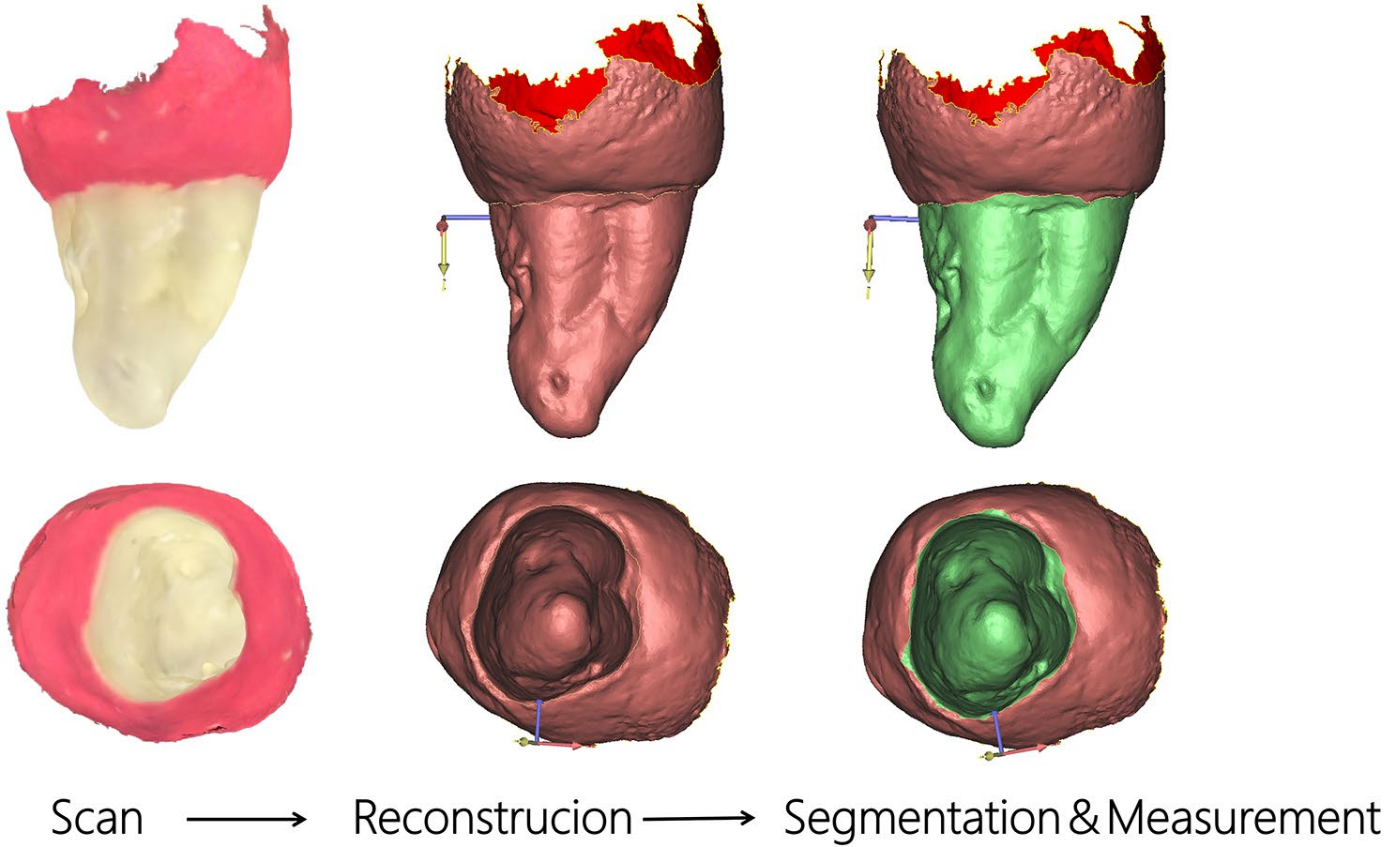
Process of optical scanning of the isolated teeth: from left to right: manual wrapping of the crown, optical scanning, 3-matic software reconstruction, manual root segmentation and calculation of the root surface area

For the control group, we used the optical scanning results of the roots of 24 teeth as a reference standard. After CBCT scanning, the clinical crowns of the 24 extracted teeth were embedded in pink clay, with the CEJ and root of the teeth exposed (Fig. 1). Each tooth specimen was scanned using an iTero® Element™ optical scanner (Align Technology, Santa Clara, California, USA) with a scanning accuracy of 0.02 mm. Optical scanner data were collected and imported into 3-matic software (version 7.0, Materialise NV, Leuven, Belgium) to construct a 3D digital model.

The roots of each tooth were manually segmented, and the RSA was measured as control data. Two dentists with more than 5 years of clinical experience performed all measurements of the digital model, and the data were obtained twice by the same investigator at an interval of two weeks. The measurement results of the two raters were averaged for data analysis. We calculated Cronbach’s alpha values to determine the internal reliability between the first and second measurements of each rater and used the intraclass correlation coefficient (ICC) to examine the inter-rater consistency.

### Segmentation and calculation of RSA in CBCT scanning

To calculate RSA, we first need to identify the tooth surface region. The non-air part is defined as the tooth part and the air part is defined as the background. Each tooth can be depicted as a connected component in 3D space. The surface region is made up of edge pixels within the connected component, where a pixel is deemed an edge pixel if at least one of its neighboring 8 pixels in 2D CBCT slices is part of the background. For this study, we regard the number of edge pixels as the surface area of the tooth region. The presence of enamel or cementum on the surface of a tooth is what distinguishes a root from a crown.

**Fig. 2:**
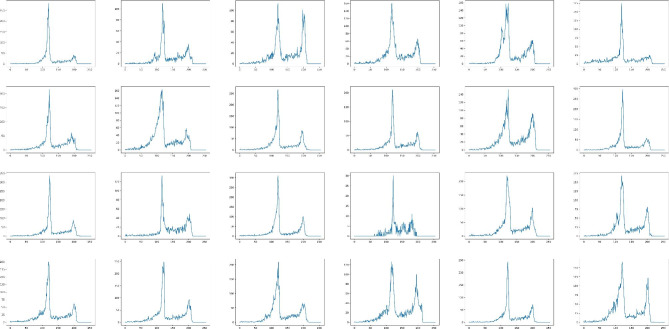
A statistical diagram of the relationship between the threshold and number of pixels at the edges of the teeth in the axial CBCT images of 24 isolated teeth. The x-axis indicates the pixel threshold values from 0 to 255, and the y-axis represents the number of pixels corresponding to each pixel threshold. There are two peaks for the threshold of each tooth, where the peak on the left side indicates the threshold of the root and the small peak on the right side indicates the threshold of the crown

Once the surface region has been identified, we distinguish between enamel and cementum by utilizing pixel thresholding, given that the pixel value of the enamel region is invariably greater than that of the cementum in CBCT images. For each of the 24 extracted teeth, we calculated the histogram of surface pixel density (Fig. 2) and discovered that each distribution exhibits two clearly separated peaks.

The presence of two distinct peaks, one on the left and one on the right, corresponds to the observed variation in pixel thresholds for cementum and enamel, respectively. This clear boundary between the peaks indirectly confirms the viability of our concept.

**Fig. 3:**
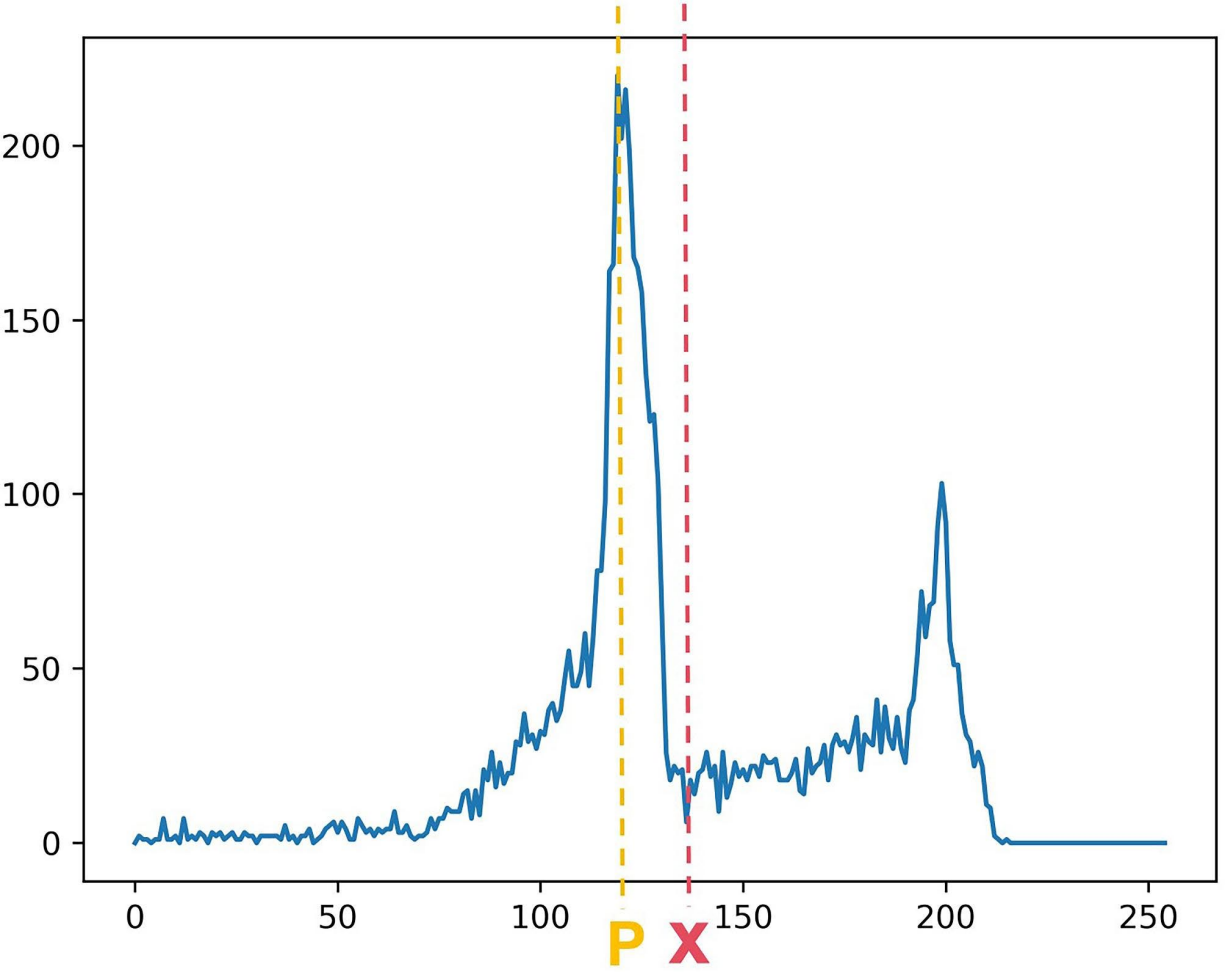
A statistical diagram of the relationship between the threshold and number of pixels at the edges of the teeth in the axial CBCT images of one isolated tooth. P represents the threshold position of the first peak in the x-axis. X represents the position of the nearest minimum threshold in the x-axis to the right of P

Given that CBCT images may exhibit different value ranges for teeth across individuals, the primary objective is to establish a definitive pixel threshold that effectively distinguishes cementum from enamel. To address this challenge, we developed a personalized dynamic approach for calculating pixel thresholds. The algorithm follows these steps:

(1) We calculated the distribution of surface pixels for each tooth, and the pixel distribution of selected teeth is shown in Fig. 2. The x-axis indicated the pixel threshold values from 0 to 255, and the y-axis represented the number of pixels corresponding to each pixel threshold. There were two peaks for the threshold of each tooth, where the peak on the left side indicated the threshold of the root and the small peak on the right side indicated the threshold of the crown.

(2) The pixel threshold of the first peak on the x-axis was denoted by *P*.

(3) We identified the location of the minimum value in the x-axis that was closest to the pixel threshold greater than *P.* The pixel threshold at this location, denoted by X, was considered the threshold required for segmentation (Fig. 3).

**Fig. 4:**
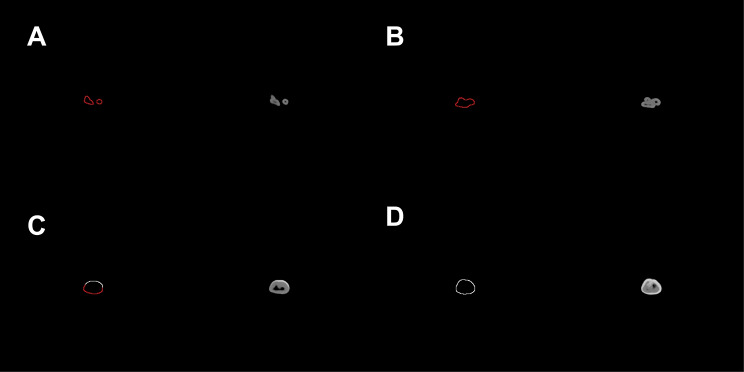
CBCT axial visualization images of isolated tooth roots segmented based on the dynamic threshold method. Images of four axial levels (A-apical 1/3, B-middle 1/3 of the root, C-root-crown junction, and D-crown level) have been selected as representative images. The red color indicates the root, and the white color indicates the crown

After identifying the pixel segmentation threshold X, we calculated the number of pixels smaller than the X threshold. Our CBCT sampling ratio was 0.3 mm = 1 pixel, so we converted the circumference calculated from the number of pixels into a physical distance (mm) as per this ratio. We calculated the RSA by first multiplying the circumference of the root by the CBCT section spacing of 0.3 mm and then adding the findings. We used this threshold X to segment the 24 isolated teeth in CBCT images, and the results are shown in Fig. 4.

### Statistical methods

We conducted data analysis using the IBM Statistical Package for Social Sciences (version 21.0, IBM Corporation, Armonk, NY, USA). We used the paired-samples *t*-test to compare differences and the Bland-Altman plot [32] to analyze the consistency between the results obtained by the two measurement methods.

## Results

Table 1 shows the RSA data of the 24 isolated teeth of the experimental and control groups, as well as the specific values obtained by the two calculation methods for each tooth and the difference and ratio between them. The visualization results of the CBCT image segmentation of tooth roots based on the dynamic threshold method are shown in Fig. 4. From the 70–80 2D axial CBCT images of each tooth, we selected the images of four axial levels (apical 1/3, middle 1/3 of the root, root-crown junction, and crown level) to show the segmentation results. The red part depicted the segmented tooth roots, and the white part depicted the segmented crowns.

**Table 1:** The results of the root surface area (RSA) of 24 isolated teeth calculated for the experimental and control groups, and the difference and ratio (RSA) between the two methods (unit: mm^2^)

No.	Experimental group	Control group	Difference	Ratio
1	284.31	290.7351	-6.43	0.98
2	295.56	301.2646	-5.70	0.98
3	260.19	256.4044	3.79	1.01
4	282.96	280.0253	2.93	1.01
5	297.72	289.6320	8.09	1.03
6	299.88	304.0388	-4.16	0.99
7	242.19	233.0616	9.13	1.04
8	216.63	220.1421	-3.51	0.98
9	371.16	380.7374	-9.58	0.97
10	276.84	277.0318	-0.19	1.00
11	212.58	208.5532	4.03	1.02
12	199.22	193.4995	5.72	1.03
13	337.86	346.7172	-8.86	0.97
14	278.55	277.9656	0.58	1.00
15	266.94	262.0894	4.85	1.02
16	277.47	280.2429	-2.77	0.99
17	361.71	355.3484	6.36	1.02
18	285.66	280.6460	5.01	1.02
19	232.92	233.1131	-0.19	1.00
20	277.65	278.5781	-0.93	1.00
21	313.92	312.9954	0.92	1.00
22	187.56	190.3053	-2.75	0.99
23	331.65	322.4861	9.16	1.03
24	212.67	210.7732	1.90	1.01

**Fig. 5:**
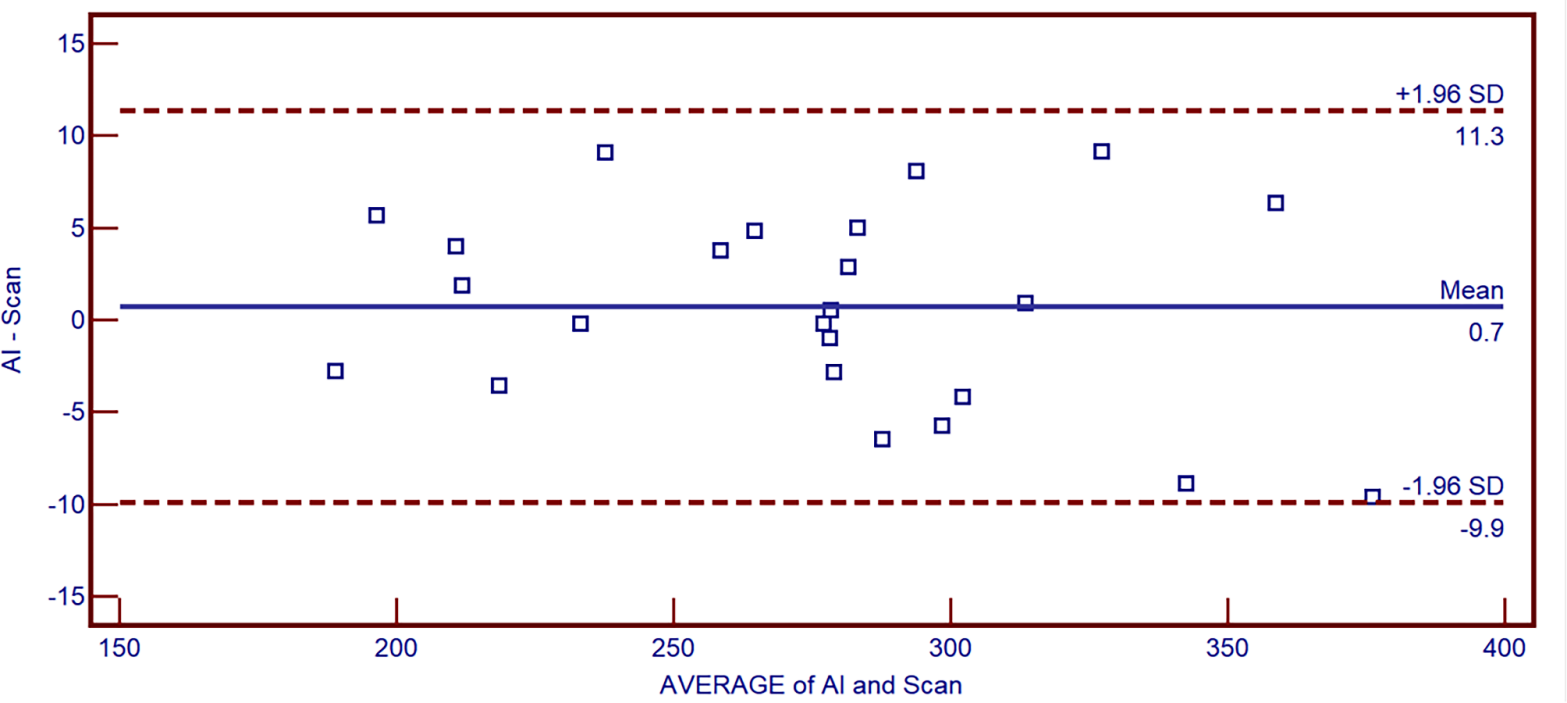
Bland-Altman plots showing the differences between the experimental and control groups. As the horizontal coordinate increases, the data are evenly and approximately distributed within ± 1.96 standard deviations of the mean, with no upward or downward trend, and the mean value of the difference is 0.7 mm^2^

Intra-rater reliability, as measured using the Cronbach’s alpha value, was 0.998, and inter-rater reliability, measured in terms of the intraclass correlation coefficient, ICC, was 0.988 (*P* < 0.001). As per the Kolmogorov-Smirnov test, the data in each group were normally distributed (*P* > 0.05). The paired t-test results showed that the difference in the calculated RSA in the experimental and control groups was 0.73 ± 5.42 mm (95% confidence interval: -1.50–3.01), which was not statistically significant (t = 1.005, *P* = 0.518 > 0.05). The correlation of paired samples was < 0.001, indicating a favorable correlation. The consistency analysis done with the Bland-Altman plot demonstrated that as the horizontal coordinate increased, the difference between the data in the experimental and control groups was evenly and approximately distributed within ± 1.96 standard deviations of the mean, and the mean value of the difference was 0.7 mm^2^, without an overall increasing or decreasing trend (Fig. 5).

## Discussion

In this study, we designed a computer algorithm based on a personalized dynamic threshold method to segment the roots of 24 isolated teeth. We compared the RSA value obtained using our algorithm with the RSA value acquired from optical scanning image reconstruction, which was utilized as the evaluation index. There were no significant discrepancies between the data obtained by the two measurement methods, and the segmentation results of the CBCT image dataset had a high degree of accuracy (t = 1.005, *P* = 0.518 > 0.05). The Bland-Altman plot showed that the measurement results had good consistency.

The accurate measurement of RSA is the basis for evaluating the grade of periodontal disease. The ratio of periodontal membrane area to the RSA can reflect the severity of periodontal disease and help determine the prognosis of teeth [1, 2]. In addition, the degree of RSA absorption has implications for the orthodontic treatment plans. Rather than just using a two-dimensional change in root length, the side effects of orthodontic treatment can be evaluated from a three-dimensional perspective based on analyzing the amount of root surface area reduction [33, 34].

Classical techniques for the measurement of the RSA for isolated teeth date back to 1940 and include film-forming, image segmentation, and mass conversion methods [6]. The rapid development of 3D image analysis and printing technology in the last 20 years has brought digital dentistry to the forefront and replaced traditional RSA measurement methods [7]. The introduction of intraoral optical scanning combined with digital imaging technology has greatly simplified the measurement of 3D data, and its accuracy has been widely verified [8–10, 13, 35]. However, the optical scanner can only scan the crown, which is the part of the tooth that is exposed to the oral cavity. Therefore, this technique is commonly used in the clinic nowadays for the purpose of assessing pre-orthodontic tooth alignment treatment plans or for restorative crown design [3]. In addition, the scan reconstruction method cannot be used to directly segment the root, and the segmentation area needs to be marked manually in advance due to the complex anatomy of the tooth. In this study, we covered the crowns with clay to increase their differentiation from the roots; we segmented the scans manually after this and used the 3-matic software for measurements. This process increases the subjective variability of the procedure and is time-consuming and labor-intensive.

There has been extensive research on segmentation in CBCT images during the last decade that has focused on the segmentation of teeth and alveolar bone, whereas tooth root segmentation has not received much attention [36]. To address the issue of classical U-net segmentation, over-segmentation, and omission of tooth roots and to enhance the root segmentation performance, Li et al [26] constructed the AttU-Net + BDC LSTM network and extracted interlayer information of tooth root sequences in CBCT images. Although they defined the root as the part of the tooth located in the alveolar bone, they have not provided any guidance on how to distinguish CEJs. In patients with periodontitis with severe alveolar bone resorption, the segmentation results are smaller than the real value of the root. Due to the CEJ being the demarcation line between the root and the crown of the tooth, [27] with no clinically significant protruding or concave structure, a separate dividing line structure cannot be observed on CBCT images. The only real distinction is that the pixel threshold in CBCT for the enamel component of the crown is significantly higher than both the cementum component of the root and the alveolar bone component. As a result, the threshold-based segmentation method remains the preferred method for tooth root segmentation. However, threshold segmentation is challenging because of the wide range of grayscale values possible in CBCT scans.

Many previous studies have reported the disadvantages of threshold segmentation [29]. For example, roots and alveolar bone can be hard to tell apart in CBCT images because of factors such as high image noise and similar thresholds in the images [28]. The relative position can affect the grayscale values even for objects with the same radiation density [31]. Grayscale values, especially in CBCT scanning, vary from instrument to instrument and from patient to patient in complex structures such as the oral cavity. Hence, a simple threshold cannot satisfy the segmentation requirement.

Accordingly, we designed a dynamic-based thresholding method to develop a personalized grayscale recognition procedure. In this study, we analyzed the pixel thresholds of the outermost ring of 24 isolated teeth and found that as the pixel threshold increased, two threshold peaks were formed: the first peak was in the cementum, that is, the tooth root region, while the second peak was in the enamel, that is, the crown region. To avoid the problem of individual threshold differences and to resolve the issue of unstandardized CBCT grayscale values, we chose the first minimum value after the first peak as the demarcation threshold for the crown and root of each tooth. The calculated results were not significantly different from the results obtained by the optical scanning of the isolated teeth, with a mean difference of 0.7 mm^2^ and excellent consistency. The computer-aided segmentation algorithm markedly shortened the time from reconstruction to manual segmentation of the root for each tooth and had fewer subjective errors in the procedure.

It has been reported that the voxel size affects the accuracy of CBCT images. When measuring linearity indexes, voxel sizes of 0.125–0.4 mm had no significant effect on the representational accuracy of teeth and alveolar bone [17, 21, 37, 38].

Notwithstanding this, volume index measurements revealed that the size of the display voxel significantly affected the imaging accuracy [38–42]. Conversely, it has also been demonstrated that lower resolutions lead to higher levels of image noise, which can reduce the accuracy of alveolar bone measurements [40, 43]. Meanwhile, higher-resolution data may require higher levels of radiation, which increases the radiation exposure of patients [44]. Unfortunately, there have not been many studies to compare surface area measurements. In this study, we used CBCT images with a voxel size of 0.3 mm, which is common in China, and found that there was no significant difference in the accuracy of the RSA data between the experimental and control groups. Therefore, a voxel size of 0.3 mm can be used to evaluate RSA data, eliminating the need for higher radiation levels for imaging thereby decreasing the risk of radiation exposure in patients.

Thresholds vary not only across patients but also between teeth in the same patient. This makes it challenging to effectively separate tooth roots using a basic threshold segmentation method. In preliminary study, we used the threshold value for the separation of crown and root in the full-mouth CBCT image of patients and found that the corresponding extracted tooth had good segmentation, but it was not applicable to all teeth. Other teeth can show over-division of the crown or over-division of the root. This indicates that there is variation in the segmentation thresholds of crowns and roots of different teeth in the same patient. The analysis of dynamic threshold done in this study is only applicable to isolated teeth. We propose to continue using the method for full-mouth CBCT images after verifying the accuracy of the dynamic threshold method for tooth root segmentation and area calculation.

Another limitation of this study is that although the confidence interval obtained by the power analysis of 24 isolated teeth verified the accuracy of the results, it is a new method, and in order to increase the reliability of RSA results obtained by radiographic segmentation and pixel counting, it requires more diverse data and larger samples. The CEJ boundaries of the teeth do not converge in every case, and each tooth group may have different configurations; thus, anatomical variations can also potentially constrain calculations. Therefore, in the future, to increase the number of teeth for verification analysis, we will continue to expand the sample size of the isolated tooth measurement and use this dynamic threshold approach for full-mouth CBCT.

## Conclusion

In this study, we designed a set of computer-aided systems based on personalized dynamic threshold segmentation of tooth roots, which yielded results with a high degree of accuracy in the root segmentation of isolated teeth when compared to optical scans. Moreover, we found that CBCT images with a voxel size of 0.3 mm were sufficient to illustrate the accuracy of the RSA of teeth. The methodology used in this study lays the groundwork for the accurate automatic segmentation of tooth roots in full-mouth CBCT images and the computation of RSA in the future.

## Data Availability

The datasets used and/or analysed during the current study available from the corresponding author on reasonable request.

## List of abbreviations

Root surface area: RSA
Cone beam computed tomography: CBCT
Cementoenamel junction: CEJ
Intraclass correlation coefficient: ICC
